# The expression of miRNAs is associated with tumour genome instability and predicts the outcome of ovarian cancer patients treated with platinum agents

**DOI:** 10.1038/s41598-017-12259-w

**Published:** 2017-11-07

**Authors:** Tianzhen Wang, Guangyu Wang, Xiaoxiao Zhang, Di Wu, Lei Yang, Guangyou Wang, Dapeng Hao

**Affiliations:** 1 Department of Pathology, Harbin Medical University, Harbin, 150081 China; 2 Department of Gastrointestinal Medical Oncology, the Affiliated Tumour Hospital of Harbin Medical University, Harbin, 150081 China; 3 College of Bioinformatics Science and Technology, Harbin Medical University, Harbin, 150081 China; 4 Department of Obstetrics and Gynecology, First Affiliated Hospital of Harbin Medical University, Harbin, 150001 China; 5 Department of Neurobiology, Harbin Medical University, Harbin, 150081 China

## Abstract

miRNAs, a class of short but stable noncoding RNA molecules, have been revealed to play important roles in the DNA damage response (DDR). However, their functions in cancer genome instability and the consequent clinical effect as the response to chemotherapy have not been fully elucidated. In this study, we utilized multidimensional TCGA data and the known miRNAs involved in DDR to identify a miRNA-regulatory network that responds to DNA damage. Additionally, based on the expression of ten miRNAs in this network, we developed a 10-miRNA-score that predicts defects in the homologous recombination (HR) pathway and genome instability in ovarian cancer. Importantly, consistent with the association between HR defects and improved response to chemotherapeutic agents, the 10-miRNA-score predicts the outcome of ovarian cancer patients treated with platinum agents, with a surprisingly better performance than the indexes of DNA damage. Therefore, our study demonstrates the implication of miRNA expression on cancer genome instability and provides an alternative method to identify DDR defects in patients who show the best effect with platinum drug treatment.

## Introduction

Genome instability is one of the hallmarks of human cancers and is often associated with cellular deficiency in the DNA damage response (DDR)^1^. Mutations and chromosomal alterations often occur in human tumours. To preserve the genomic integrity, cells have evolved an elaborate cellular system to sense and repair DNA lesions^2^. DNA double-strand breaks (DSBs) are one of the most severe types of DNA damage and are repaired by the error-free homologous recombination (HR) or error-prone non-homologous end joining (NHEJ). Other types of DNA damage are processed through mismatch repair (MMR), base excision repair (BER) and nucleotide excision repair (NER)^3^. Hereditary and somatic defects in DDR genes could lead to genome instability and various human cancers. For example, mutations in NER genes predispose to skin cancer, and mutations in HR genes predispose to various cancers, including ovarian and breast cancer, lymphomas and leukaemia^4–6^. However, high-throughput sequencing studies in recent years have revealed that genome instability in most sporadic human cancers is not due to mutations targeting caretaker genes in these pathways, raising the need to consider aberrant post-transcriptional regulation that can also inactivate DDR genes^1^.

miRNAs that are involved in the highly coordinated network of DDR processes might play important roles during the development of genome instability and might determine the cellular response to DNA damaging agents^7^. Increasing evidence has shown that cells deficient for miRNA biogenesis have abnormal cell cycle checkpoints and DNA repair^8^. Additionally, many miRNAs, such as miR-24, miR-21, miR-146, miR-421 and miR-373, have been implicated in DDR^9^. miR-24 attenuates H2AX expression, resulting in higher sensitivity to IR and reduced DNA repair ability^10^. miR-421 down-regulates ATM, leading to altered cell cycle checkpoint and increased radio-sensitivity in cells^11^. miR-146 targets BRCA1 and is involved in the response to DSBs^12^. These findings demonstrate that DDR genes are regulated by miRNAs and suggest that the aberrant expression of miRNAs would affect the DDR process. In fact, several recent studies have revealed that aberrant expression of miRNAs in human cancer affects the sensitivity of DNA damage-based therapy. For example, overexpression of miR-151 by the gain of chromosome 8q24.3 facilitates tumour cell migration and spreading through the downregulating DDR gene RhoGDIA^13^. The miR-183-96-182 cluster, whose expression is increased in many human cancers, increases the sensitivity to platinum agents and PARP inhibitors by downregulating RAD51, REV1 and BRCA1^14,15^.

Due to the key role of DDR in sensing and repairing DNA damage, the combined use of agents targeting DDR and DNA damaging agents is becoming a very attractive strategy in cancer treatment^16^. For example, the standard treatment of epithelial ovarian cancer, a cancer type characterized by frequent HR deficiency, is aggressive surgical debunking, followed by platinum-based chemotherapy, delivered concurrently with other agents (i.e., taxane). The chemotherapy is most effective with patients exhibiting severe DDR deficiency. Improved survival has been observed in ovarian cancer patients with mutations of *BRCA1* or *BRCA2* when treated with platinum agents^17–19^. However, there are various genomic alterations of DDR genes that may cause different extents of DDR deficiency, whereas a standardized signature for assessing the severity of DDR deficiency has not been established. Realizing that caretaker genes are under exquisite regulation by miRNAs, we utilized multidimensional TCGA data and the known miRNAs involved in DDR to identify the miRNA-regulatory network responding to DNA damage in ovarian cancer. Based on the expression of these miRNAs, we have developed a miRNA-score that is associated with genome instability and predicts the outcome of ovarian cancer. We found that our miRNA-score reflects HR deficiency and can identify chemo-sensitive samples with or without mutations of caretaker genes.

## Results

### Construction of a miRNA-regulatory network involved in DDR

**Figure 1:**
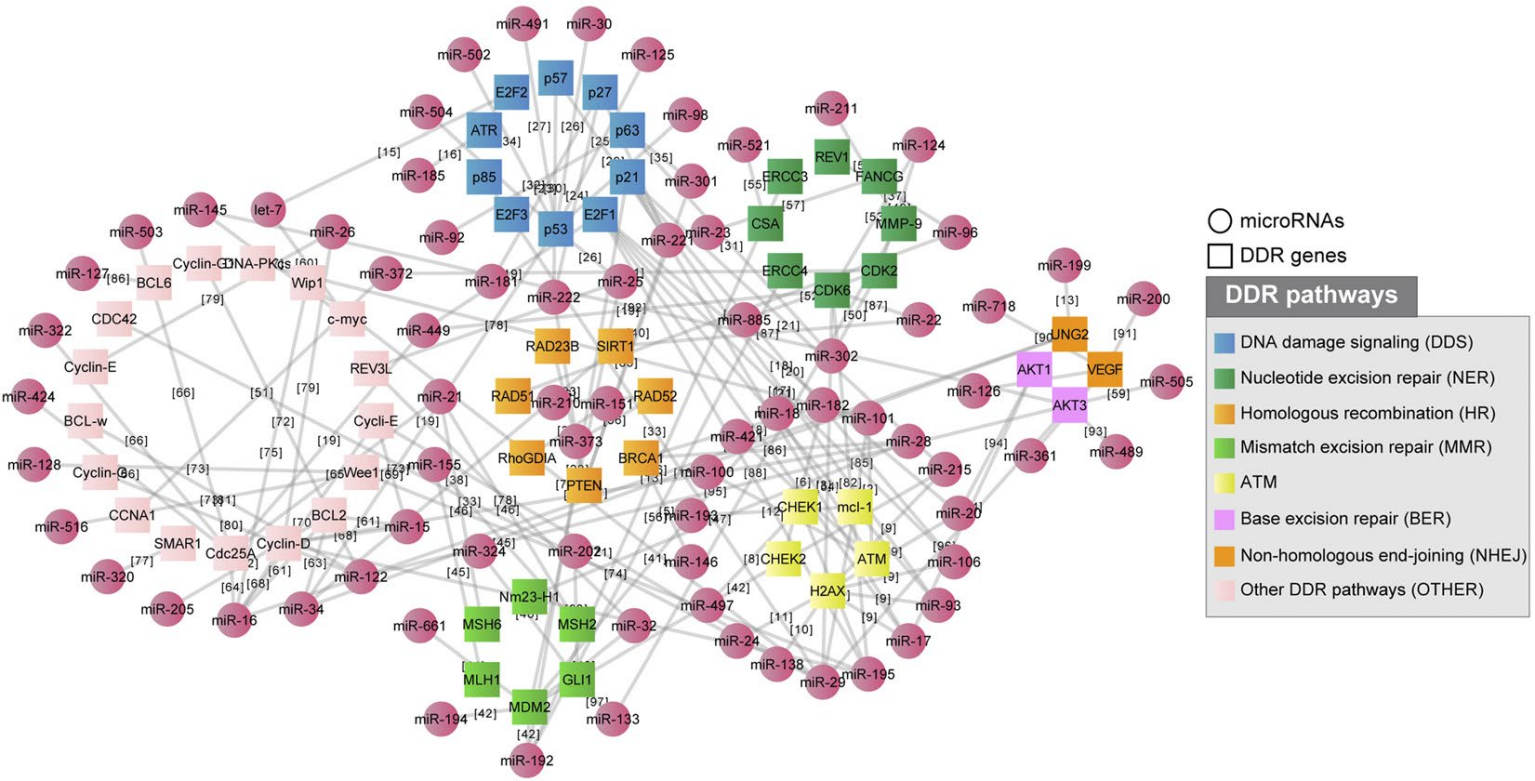
Construction of a miRNA-regulatory network involved in the DDR consisting of 142 miRNA-target interactions between 75 miRNAs and 55 DDR genes.

To collect a high-quality set of miRNAs in the regulatory network involved in DDR, we developed the following strategy: (1) we obtained 522 DDR genes involved in the “cellular response to DNA damage stimulus” according to Gene Ontology annotation; (2) we downloaded all the miRNA-gene interactions between miRNAs and the 522 DDR genes from the starBase 2.0 database and only kept the interactions supported by both CLIP-Seq experiments^20^ and at least one of the five miRNA prediction software programs (TargetScan, PicTar, PITA, miRanda and RNA22); (3) we searched Pubmed for each interaction using the name of the miRNA and gene symbol as keywords, and manually checked the publications to ensure that the target gene expression is repressed by the miRNA. The resulting miRNA-regulatory network involved in DDR consists of 142 miRNA-target interactions between 75 miRNAs and 55 DDR genes. Specific information, including the miRNA-gene interactions, DNA repair pathway of the target gene and supporting references, is provided in Supplementary Table 1 and are plotted in Fig. 1.

All the DDR genes have been classified as DNA damage signalling (DDS) genes or genes belonging to one of the following DNA repair pathways: HR, NHEJ, MMR, NER and BER. Genes involved in other pathways—i.e., cross-link repair, RecQ helicase pathway and translation synthesis—were clustered together into one group due to the small number of genes within each group. For genes involved in multiple pathways, such as ATR and ATM, we classified them into the most appropriate pathway according to our knowledge. Thus, miR-29 and miR-16 were identified as the most connected miRNAs, and *TP53* and its inhibitor *MDM2* were found to be the most regulated gene targets. Fourteen genes of this network, including ATM, CHEK1, CHEK2, H2AX, MCL-L, BRCA1, RAD23B, RAD51, RAD52, SIRT1, RhoGDIA, PTEN, AKT1 and AKT3, are in pathways known to be activated in response to platinum^19^. To further demonstrate the importance of this network in the DDR, we investigated the expression change of genes in the network upon DNA damage. We found that approximately 40% of these genes showed a significant expression change in 36M2 ovarian cancer cells after treatment with carboplatin, and approximately 60% of these genes showed a significant expression change in MCF7 cells after treatment with doxorubicin (See Supplementary Table 2)^21,22^. miRNAs in this network also show significant change in the DDR. For example, Blume, C.J. *et al*. reported 41 significant miRNAs regulated after treatment with 24 h of irradiation versus no treatment in 68 paired primary samples of chronic lymphocytic leukaemia (CCL) by analysing 1,405 candidate miRNAs^23^. Notably, 23 of these 41 differentially expressed miRNAs are included in our miRNA-regulatory network, which is highly enriched (p < 0.05, 41 of 1405 vs. 23 of 75, Chi-squared test). This includes the key p53 target miR-34 that showed the most dramatic expression change upon DNA damage. It has been shown that miR-34 may also be involved in the DDR by targeting *SIRT1* and *UNG2* ^24,25^.

### Identification of miRNAs involved in genome instability in ovarian cancer

Genome instability manifests in various forms, such as chromosomal abnormality, somatic mutation and microsatellite instability. Different measurements of genome instability, including the total aberration index (TAI) of copy number variation and loss of heterozygosity (LOH) frequency, have been used in previous studies^26–28^. Here, we propose that the mutation frequency is a quantified measurement of genome instability regarding ovarian cancer, which is characterized by frequent HR deficiency^29^. To verify this, we compared tumours with known BRCA1/2 deficiency and wild-type tumours using 319 tumours with level 3 mutation data of the TCGA dataset. Four overall indexes of genome instability, the fraction of the altered genome, TAI, frequency of LOH and frequency of somatic mutation, were analysed, whereas only the LOH and mutation frequencies showed a significant difference between the two groups of tumours in the TCGA dataset. The frequency of mutation is significantly correlated with the frequency of LOH but shows a broader distribution that is more suitable to further stratify different tumours (Supplementary Figure 1). Moreover, the mutation frequency is more predictive of BRCA deficiency and clinical outcome than LOH.

We then evaluated the association between the expression of miRNAs and mutation frequency of the cancer genome as an index of genome instability. By analysing the expression of 723 human miRNAs across 319 TCGA high-grade advanced-stage ovarian tumours, we identified 17 miRNAs whose expression showed a significant association with the frequency of mutation (Mann-Whitney U test; p < 0.01; one sided), with ten of them in our miRNA-regulatory network (significance of enrichment, p < 0.01; Fisher exact test; 10 of 75 miRNAs in Fig. 1 versus 17 of 723 analysed miRNAs), including miR-151, miR-301b, miR-505*, miR-324, miR-502 and miR-421, for which higher expression is associated with a high frequency of mutation, and let-7a*, miR-320, miR-146a* and miR-193a, for which lower expression is associated with a high frequency of mutation.

**Figure 2:**
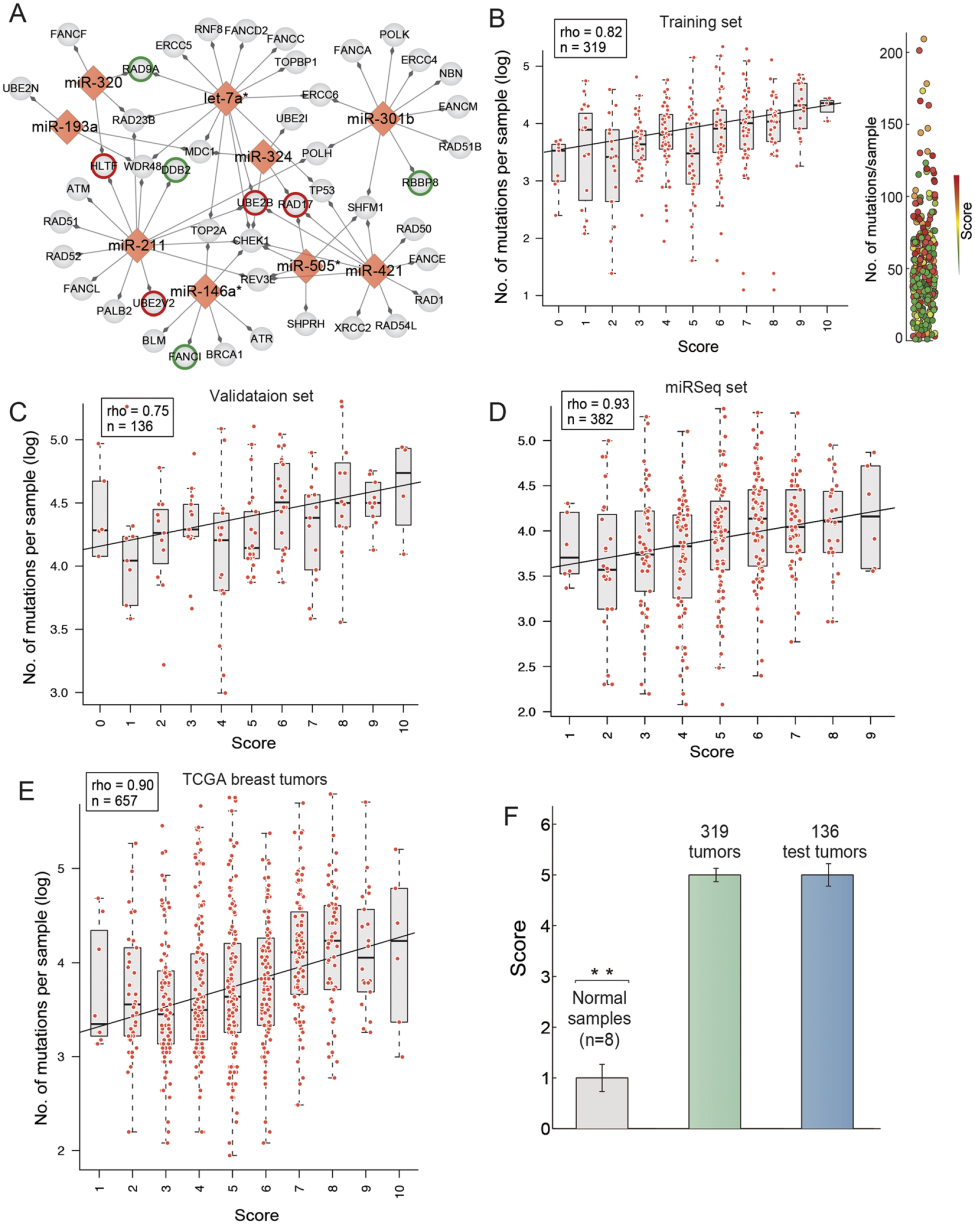
The 10-miRNA-score predicts genome instability in ovarian cancer. (**A**) The predicted targets of the ten miRNAs correlated with genome instability are enriched in the DDR genes responsive to platinum. miRNA miR-151 and miR-502 are not shown here because no platinum-responsive DDR genes were found. The coloured border indicates the prognostic genes of ovarian cancer (green for favourable outcome and red for poor outcome) (**B**). The correlation between the 10-miRNA-score an level of DNA damage in the training set of ovarian cancer. Shown on the right panel is a visual illustration of the distribution for tumours with different numbers of mutations and 10-miRNA-scores. (**C**) Validation of the correlation between the 10-miRNA-score and DNA damage level in an independent dataset of ovarian cancers. (**D**) Validation of the correlation between the 10-miRNA-score and DNA damage level using miRNA-Seq data. (**E**) The 10-miRNA-score also shows a significant correlation with genome instability in breast cancer. (**F**) The 10-miRNA-score of normal controls was significantly lower than that of tumour samples of ovarian cancer (p < 0.01 compared with tumours in the training set and the test set).

To highlight the potential clinical relevance of these miRNAs in the treatment of ovarian cancer, we collected 80 DNA repair genes from previous studies that showed a response to platinum drugs (Supplementary Table 3). Of these collected genes, 40 are predicted targets of eight of the ten miRNAs. Compared with the background of the human genome, the 6,215 potential targets of the 10 miRNAs that are supported by any of the five abovementioned miRNA prediction algorithms were significantly enriched in platinum-responsive genes (fold enrichment: 2.4; hypergeometric test; p < 10^−3^). We also found that the targets of miR-211, which is not included in the curated miRNA-regulatory network, are significantly enriched in DDR genes (GO function enrichment analysis by David; FDR < 0.05). In addition, the higher expression of miR-211 is associated with increased mutation number (Mann-Whitney U test, p = 0.02) in ovarian cancer, which suggests that this miRNA is likely involved in genome instability regulation. The miRNA-target regulations between platinum-responsive genes and the 8 miRNAs, as well as miR-211, are plotted in Fig. 2A. Interestingly, many of the target genes in Fig. 2A are prognostic genes of ovarian cancer (Supplementary Figure 2 and Supplementary Table 3).

### The 10-miRNA expression score predicts genome instability

To develop a simple miRNA-based signature with strong predicting power of genome instability, we constructed a 10-miRNA-score for each patient, devised by simply adding the number of positively correlated miRNAs (miR-151, miR-301b, miR-505*, miR-324, miR-502 and miR-421) of a patient that show higher expression than the median value across the dataset and the number of negatively correlated miRNAs (let-7a*, miR-320, miR-146a* and miR-193a) that show lower expression than the median value across the dataset. In other words, for each patient, we assigned a point for each of the 6 miRNAs (miR-151, miR-301b, miR-505*, miR-324, miR-502 and miR-421) if the patient showed a higher than median expression of miRNA and assigned a point for each of the 4 miRNAs (let-7a*, miR-320, miR-146a* and miR-193a) if the patient showed a lower than median expression of miRNA, and then summarized these points as the 10-miRNA-score. As expected, the 10-miRNA-score was strongly correlated with the level of DNA mutation in the cancer genome of the training set of 319 ovarian cancers (Spearman correlation between the 10-miRNA score and median level of mutation frequency, rho = 0.82; p = 0.005; Fig. 2B).

To assess the predicting power of the miRNA-based signature, we applied the 10-miRNA-score to an independent set of 136 TCGA ovarian cancer samples with provided raw mutation file and miRNA microarray expression, but not included in the level 3 validated data. A significant correlation was also observed in this dataset (rho = 0.75, p = 0.01; Fig. 2C). We further validated the association by constructing the signature using expression detected by miRNA-Seq. In total, 382 ovarian cancer samples provided with miRSeq profiles and MAF files were analysed. Although the miRNA-Seq and miRNA array for TCGA ovarian cancer were found to be poorly correlated^30^, the miRNA-Seq-based 10-miRNA-score was significantly correlated with the level of DNA mutation (rho = 0.93; p < 0.001; Fig. 2D). The relatively higher variation of the association in the validation set is likely due to the smaller number of samples. It was reported that HR deficiency also underlies the development of some breast cancers^31^; thus, we tested the association using 657 TCGA breast tumours for which both miRSeq data and mutation data were available. We found that the 10-miRNA-score was also significantly correlated with gene mutations in breast cancer (rho = 0.90; p < 0.001; Fig. 2E), suggesting that the signature predicting genome instability is not limited to ovarian cancer. Additionally, we calculated the miRNA-score of normal control tissues for which we assumed had relatively intact DDR ability and stable genomes. We found that the 10-miRNA-score of normal controls was dramatically lower than that of tumour samples (Mann-Whitney U test, p < 0.001, Fig. 2F). Taken together, these results demonstrate the capability of the expression of miRNAs in predicting cancer genome instability.

To highlight the importance of using miRNAs in the miRNA-regulatory network to construct the signature, we tested the 7 miRNAs not in our network to construct it. Although the signature based on these 7 miRNAs showed a strong association with the gene mutations in the training set, it lost the ability to predict gene mutations in the validation set.

### The 10-miRNA-score predicts defects in the HR pathway

**Figure 3:**
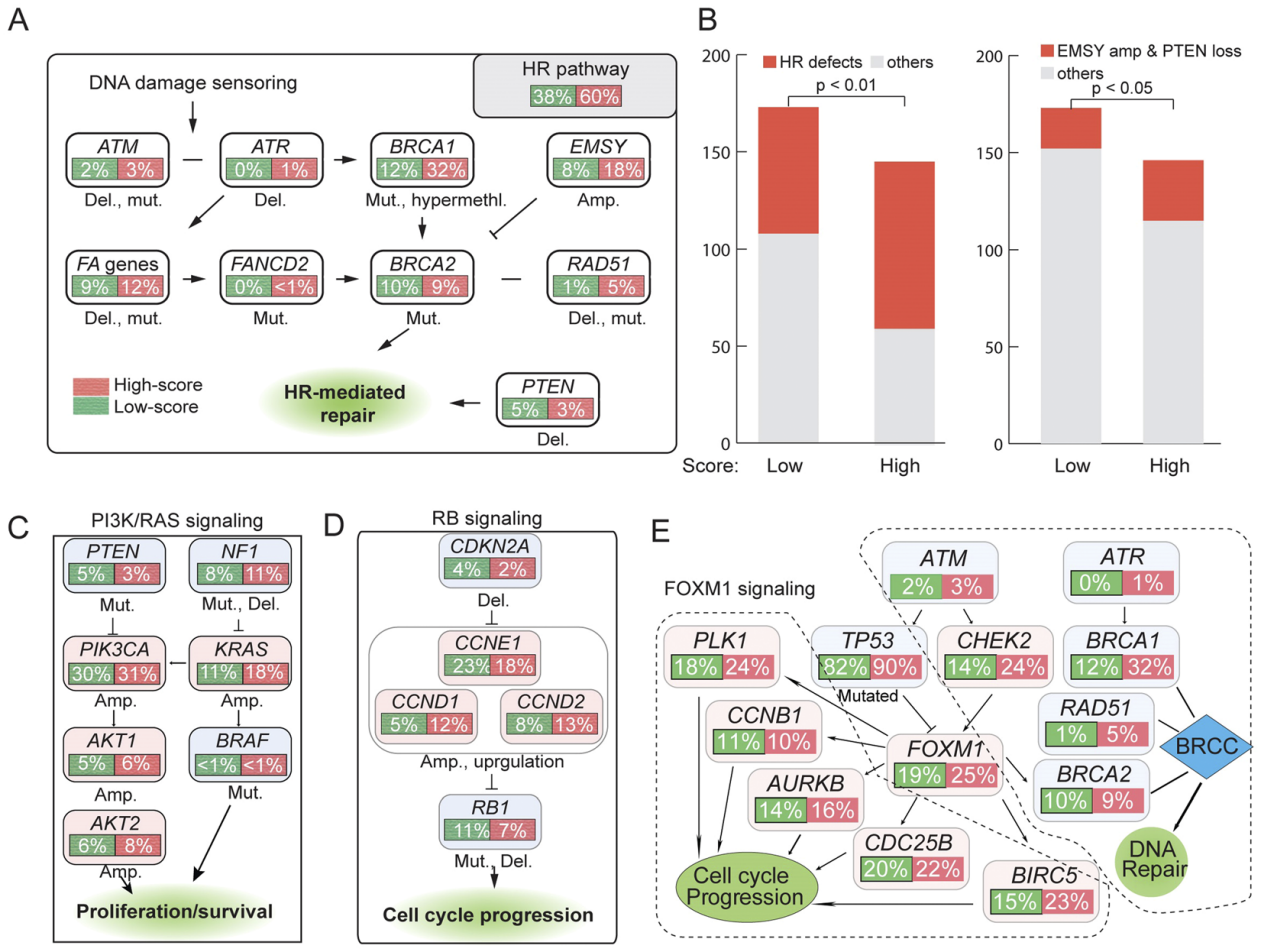
The 10-miRNA-score predicts defects in the HR pathway in ovarian cancer. (**A**) The genomic alterations in HR pathway related genes between the high 10-miRNA-score (green) and low 10-miRNA-score (red) groups in ovarian cancer. (**B**) Frequency of HR defects. The left panel shows the frequency of HR genes in the high 10-miRNA-score group compared with that in the low 10-miRNA-score group. The right panel shows the number of tumours with amplification of EMSY or copy number loss of PTEN in the high 10-miRNA-score and low 10-miRNA-score groups. (**C**,**D** and **E**) Alterations of the PI3K/RAS (**C**), RB (**D**) and FOXM1 (**E**) signalling pathways that are commonly altered in ovarian cancer. Alterations are defined by somatic mutations, amplifications and up- or downregulated gene expressions. The background colour of each node represents activation (light blue) or inactivation (light red). The percentages of alteration are in green frames for the low 10-miRNA-score group or in red frames for the high 10-miRNA-score group.

The TCGA group has identified several pathways that are commonly altered in ovarian cancer, including the RB1 signalling pathway, PI3K/RAS signalling pathway, FOXM1 transcription factor network and HR pathways^32^. To investigate whether the 10-miRNA-score was associated with the alterations of these pathways, we categorized ovarian cancer patients into the low 10-miRNA-score group (0–5) and high 10-miRNA-score group (6–10). Analysis of the frequency of these pathways harbouring mutations, copy number changes or epigenetic silencing events showed that the HR pathway was the most significantly altered pathway in the high-versus-low 10-miRNA-score group. Genomic alterations in HR-related genes that might have clinical relevance in ovarian cancer are shown in Fig. 3A. Overall, HR defects were present in 60% (86/146) of patients in the high 10-miRNA-score group and 38% (65/173) of patients in the low 10-miRNA-score group (Fisher exact test, p < 0.01) (Fig. 3B). Defects in BRCA1/2 genes, defined as mutated or methylated BRCA1 and mutated BRCA2, and widely used to identify HR deficiency, were found in 40% and 21% of patients in the high and low 10-miRNA-score group, respectively (Fisher exact test, p < 0.01). Amplification of EMSY and homozygous deletion of PTEN, which have been shown to be associated with HR deficiency, were found in 21% (31/146) and 12% (21/173) of patients in the high and low 10-miRNA-score group, respectively (Fisher exact test; p = 0.03; Fig. 3B). These results indicate that the signature identified tumours with known HR deficiencies caused by mutations or other genomic alterations.

However, we did not observe significant differences in the genomic alterations between the high- and low 10-miRNA-score groups for the PI3K/RAS signalling pathway and RB signalling pathway that control tumour cell proliferation (Fig. 3C,D). In normal cells, increased genome instability could inhibit *FOXM1* signalling though *TP53* to control cell proliferation. However, in ovarian cancer, a high frequency of *TP53* mutation (87%) causes the constitutive overexpression of FOXM1 and its target genes. Despite the different frequencies of DDR defects and different levels of genome instability between the high- and low 10-miRNA-score groups, we did not find a significant difference in the upregulation of FOXM1-related genes (Fig. 3E). These results suggest that tumours of the high- and low 10-miRNA-score groups are unlikely to show dramatic differences in terms of cell proliferation.

### The signature predicts the outcome of ovarian cancer patients treated with platinum agents

**Figure 4:**
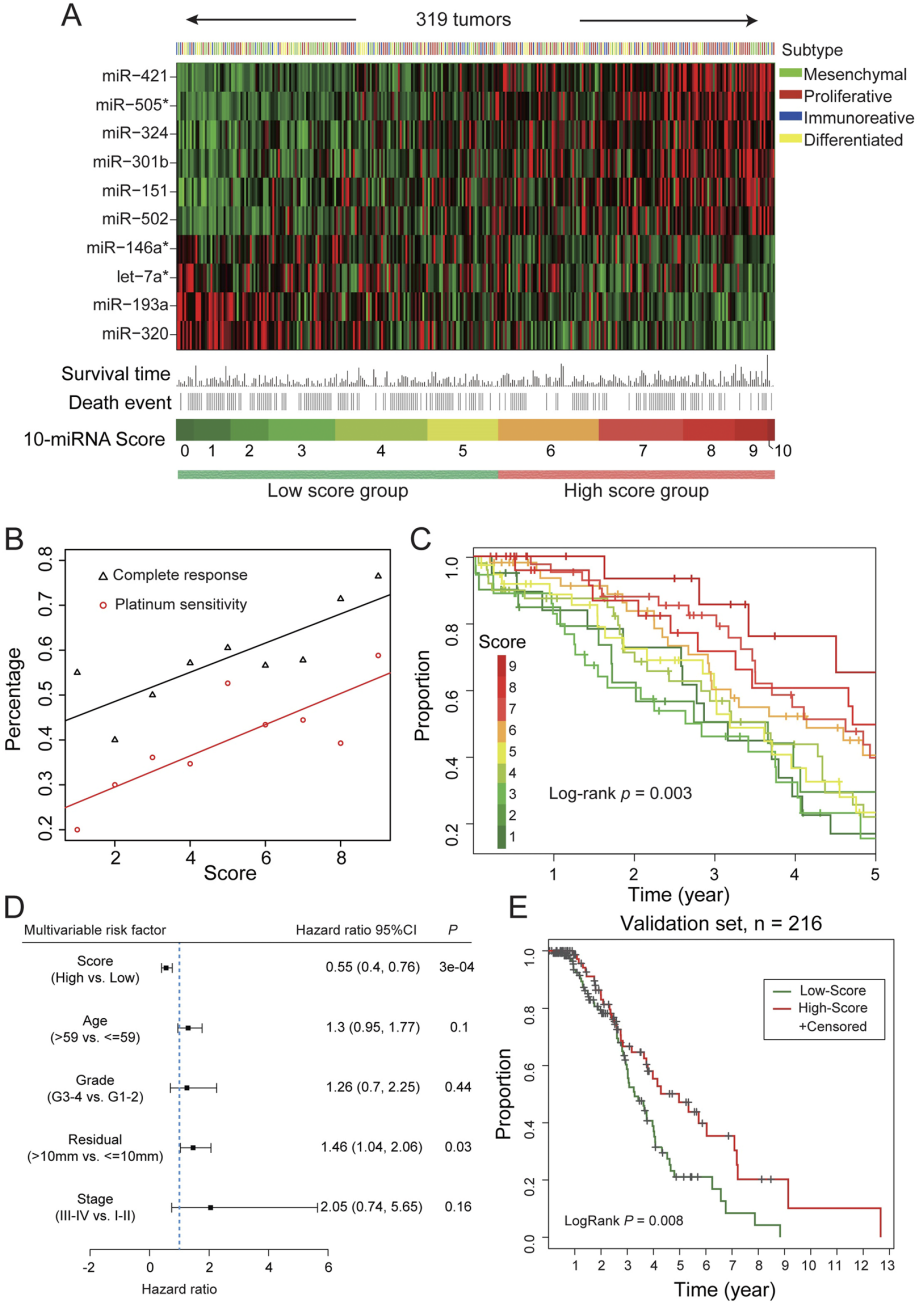
The 10-miRNA-score predicts the outcome of ovarian cancer patients treated with platinum agents. (**A**) Heatmap of the expression of the 10 miRNAs and the association with clinical information, miRNA-score and molecular subtypes for 319 TCGA ovarian cancer patients. Tumours are ordered by the 10-miRNA-score. (**B**) The percentage of platinum-sensitive patients and percentage of patients achieving CR are associated with the increasing miRNA-score. (**C**) Improved survival is significantly associated with increasing 10-miRNA-score. (**D**) Multivariable Cox proportional hazards regression analysis of the 10-miRNA-score and the clinical parameters including age, FIGO stage, histological grade and size of surgical debulking. 10-miRNA-score is treated as a categorical variable (high vs. low). (**E**) Validation of the association between the 10-miRNA-score and survival using 216 independent TCGA ovarian cancer patients treated with platinum agents. These patients are not provided with level 3 mutation data but are provided with miRNA expression and outcome information.

The expression of the ten miRNAs, 10-miRNA-score and clinical information for the 319 ovarian cancer patients are shown in Fig. 4A. The samples were classified into four subtypes (mesenchymal, proliferative, immunoreactive and differentiated) based on the mRNA expression of characteristic genes as described in a previous study. Briefly, the mesenchymal subtype is characterized by high expression of stromal components. The proliferative subtype is characterized by high expression of proliferation markers and low expression of ovarian tumour markers. The immunoreactive subtype was defined by high expression of T-cell chemokine ligands and their receptors. The differentiated subtype represents a more mature stage of development and is characterized by high expression of ovarian tumour markers^32^. As seen, the low 10-miRNA-score group contains more tumours of the mesenchymal subtype, whereas the high 10-miRNA-score group contains more tumours of the proliferative subtype. Overall, 40% (129) of the 319 ovarian cancer patients were platinum sensitive, and 58% (186) of patients achieved a complete response (CR) to platinum-based chemotherapy. Plotting the percentage of platinum-sensitive patients and percentage of patients achieving CR against an increasing 10-miRNA-score revealed strong correlations (r = 0.82 and r = 0.83, respectively, p < 0.01; Fig. 4B), suggesting that the signature could stratify tumours into groups with different efficiencies in the DDR. A high 10-miRNA-score associated with extreme genome instability indicates severe deficiencies in the DDR, explaining the sensitive response to chemotherapy.

Consequently, the 10-miRNA-score is significantly associated with favourable survival (log-rank p = 0.003; Fig. 4C): the 5-year survival rate of patients with the lowest 10-miRNA-score is only 16.8%, whereas the rate of patients with the highest 10-miRNA-score is 65.2%. A similar result was also seen between the miRNA-score and progression-free survival (PFS) (Supplementary Figure 3). When categorizing the 10-miRNA-scores into two groups, the high 10-miRNA-score group showed significantly longer overall survival than the low 10-miRNA-score group in the univariate (hazard ratio = 0.57 [95%CI, 0.42–0.78], p = 0.0005) and multivariate Cox regression analyses (hazard ratio = 0.55, p = 0.0003) (Fig. 4D), and the 10-miRNA-score outperformed all pretreatment clinical factors, including age, FIGO stage, histological grade and size of surgical debulking (Fig. 4D). The median OS in the high- and low 10-miRNA-score groups were 4.71 years (95% CI = 3.71 to 5.55 years) and 3.2 years (95% CI = 2.84 to 3.76 years), respectively. The association between the 10-miRNA-score and survival was also validated using 216 independent TCGA ovarian cancer patients treated with platinum agents, for which the miRNA expression data and clinical information were accessible (high vs. low 10-miRNA-score, log-rank p = 0.008; Fig. 4E). Interestingly, as shown in Fig. 2A, RAD17 is regulated by three miRNAs (miR-421, miR505* and miR-324) positively associated with genome instability and is a prognostic gene predicting a favourable outcome, whereas RAD9A is regulated by two miRNAs (let-7* and miR-320) negatively associated with genome instability and predicts a poor outcome.

### The prognostic value of the signature is better than genomic aberrations and known HR defects

**Figure 5:**
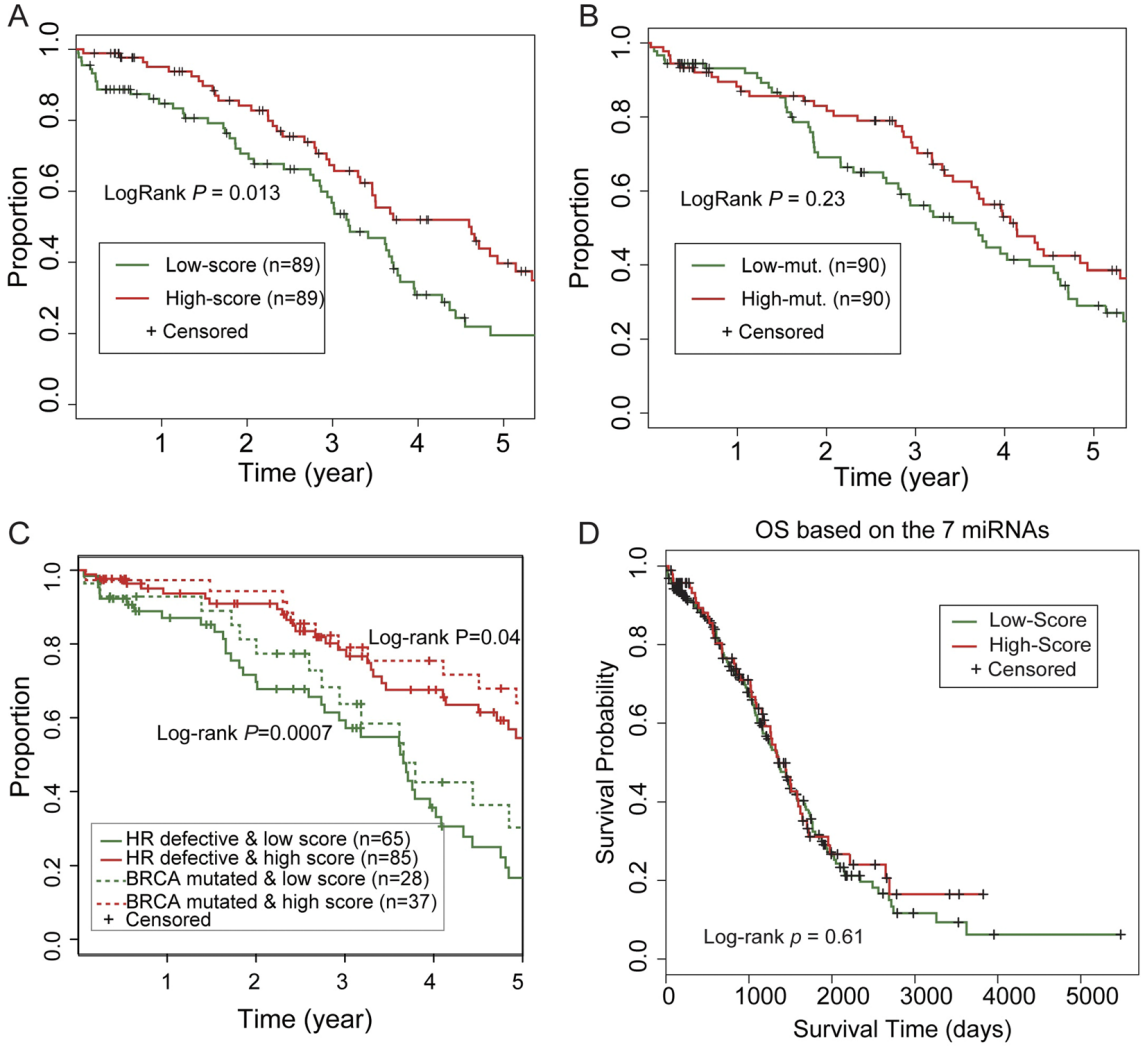
The 10-miRNA-score is a better predictor for the outcome of ovarian cancer patients treated with platinum agents than indexes of DNA damage. (**A**) The patients with different 10-miRNA-scores but similar number of mutations still show significantly different outcomes. (**B**) The number of mutations is not significantly associated with survival differences after controlling for the 10-miRNA-score. (**C**) A high 10-miRNA-score was significantly associated with improved survival in HR-defective patients or in BRCA1/2-mutated patients. (**D**) The alternate 7-miRNA-score could not significantly differentiate the survival time of ovarian cancer patients.

To investigate whether the 10-miRNA-score outperformed the frequency of genomic mutation in predicting survival, we developed a sample selection strategy to divide 178 patients into the high 10-miRNA-score and low 10-miRNA-score groups with almost the same distribution of mutation frequency (Mann-Whitney U test; p = 0.9) and divided 180 patients into the high-mutation and low-mutation groups with almost the same distribution of the 10-miRNA-score (Mann-Whitney U test; p = 0.99). We found that patients within different 10-miRNA-score groups with a similar genomic mutation frequency still showed significantly different outcomes (log-rank p = 0.01; Fig. 5A). However, groups with different genomic mutation frequencies but with a similar distribution of 10-miRNA-score were not associated with a significant difference in outcomes (log-rank p = 0.23; Fig. 5B). This indicates that the molecular signature is a better prognostic factor than directly measuring genome instability.

Moreover, the signature not only predicts the outcome for patients without known HR gene defects but also further stratifies patients with known HR defects, including those with *BRCA1/2* mutations, into groups with different outcomes. A high 10-miRNA-score was significantly associated with improved survival among patients with genomic alterations on core HR genes, and among *BRCA1/2* mutated patients (log-rank p < 0.01 and p < 0.05, respectively; Fig. 5C). To verify the importance of a molecular signature using miRNAs in the miRNA-regulatory network, we repeated our analyses using an alternate miRNA-scoring system by the seven miRNAs with expression correlated with genomic mutation but not included in our miRNA-regulatory network. Application of this alternate 7-miRNA-score to the TCGA ovarian cancer dataset revealed no significant difference in survival between the high 7-miRNA-score group and low 7-miRNA-score group (log-rank p = 0.61; Fig. 5D). Taken together, we showed that the miRNA expression signature, derived from a curated miRNA-regulatory DDR network, could stratify the DNA repair ability of tumours and predict the survival of ovarian cancer patients treated with platinum agents.

## Discussion

Genome instability resulting from the deficiency of DDR is a double-edged sword: on the one hand, it contributes to the tumourigenesis and progression of human malignant tumours^1^; however, on the other hand, it brings benefits for cancer patients treated with DNA damaging agents^16^. Therefore, the detection of DDR deficiency may be helpful to predict the chemotherapeutic effects of human malignant tumours. This is especially the case in epithelial ovarian cancer, for which at least half of patients have deficiencies in HR repair, and the standard treatment is platinum-based chemotherapy^32^. Until now, there are more than ten individual studies claiming to successfully establish prognostic models based on the expression of genes. The molecular signatures have been established based on the expression of genes enriched in extracellular matrix pathways, angiogenesis and DNA damage repair^33^. In particular, a molecular signature based on the expression of 23 genes that are involved in the platinum-induced DNA damage repair pathway was generated to predict the outcomes of ovarian cancer patients treated with platinum-based chemotherapy^19^. However, a recent meta-analysis, aimed to identify successfully published prognostic gene signatures, demonstrated that the strategy to construct molecular signatures based on DDR genes has limited performance and still requires improvement to be of clinical value^33^. One of the reasons could be the high mutation frequency of these DNA repair genes, such that the expression of a functionally inactivated gene has impaired power of predicting DNA repair ability.

MicroRNAs (miRNAs) are small non-coding RNA molecules that regulate gene expression at the post-transcriptional level. miRNAs possess multiple attractive features to allow clinical use, such as in the diagnosis and prognosis of diseases. These attractive features include stability and resistance to degradation, relatively easy detection and quantitation. Additionally, because they are small and regulate gene expression via a “seed sequence” only consisting of 6 to 7 nucleotides, a miRNA could target multiple genes. For example, let-7 has been revealed to play central roles in regulating cell metabolism via targeting multiple genes involved in this process, such as InsR, IGFR, IRS, PDK1, AKT and Rictor^34^, implying that it is possible to identify miRNA regulating networks in participating certain cellular process. Because of the active involvement of miRNAs in the DDR and ability to target multiple genes to regulate DDR^14,15^, modulating miRNA expression may be a promising strategy to identify DDR deficiency and predicting the efficacy of conventional cancer therapy with DNA damaging agents. Indeed, several studies have identified some miRNAs targeting the DDR process in ovarian cancer. For instance, miR-214 induces chromosomal instability in ovarian cancer via the downregulation of ubiquitin ligase RNF8, which is necessary for γH2AX to recruit DNA repair proteins to DNA damage sites^35^. miR-107 and miR-222 sensitizes tumour cells to the PARP inhibitor olaparib by targeting the expression of RAD51 and, thus, impairing the HR pathway and repressing the DDR^36^. However, a powerful prognostic model based on miRNA expression has not yet been constructed.

In this study, we first curated a miRNA-target list involved in the DDR, providing a hypothesis-driven signature using the expression of known miRNAs implicated in the regulation of the DDR. Unlike most previous studies, we deliberately avoided machine learning methods and the clinical information of patients to develop the signature to overcome the over-fitting that is commonly seen in most prognostic models^37^. We hypothesize that the deficiency in the DNA damage response leads to genome instability that can be measured by high-throughput sequencing technology. In ovarian cancer, chromosome instability based on copy number variation has been successfully applied to identify patients responsive to chemotherapy in recent studies^26–28,38^. In this study, we propose that the accumulation of somatic mutations is probably the most direct index of HR deficiency in ovarian cancer. This is also supported by a recent whole-genome sequencing study of ovarian cancer that revealed that chemo-sensitive tumours showed obviously more somatic mutations than chemo-resistant tumours^39^. The accumulation of indels (insertion-deletion) might be a more direct reflection of HR deficiency. However, according to the TCGA data, ovarian cancer only showed on average 2.3 indels per tumour, and approximately 40% of tumours do not show any indel at all, thus restricting its predictive power.

To avoid the rising concern of reproducibility that stems from the incorporation of unrelated genes^37^, all of the selected miRNAs for further study were examined prospectively by text-mining and extensive literature studies. Next, 10 miRNAs were extracted and used to generate a 10-miRNA-score in a learning process independent of any connections with clinical data. We demonstrated that this 10-miRNA-score not only predicts the genome instability and HR deficiency in ovarian cancer but also predicts the outcome of ovarian cancer patients treated with platinum agents. Thus, this miRNA-regulatory network involved in the DDR can serve as a useful resource to search for better biomarkers based on DDR deficiency.

In conclusion, this study strongly suggested that the 10-miRNA-score based on the expression of ten extensively studied miRNAs is correlated with the severity of HR deficiency and provides a prognostic tool to predict the chemotherapeutic effect of ovarian tumours to platinum.

## Materials and Methods

### Datasets and data processing

In this study, 602 ovarian cancer samples and eight normal control tissues of ovarian cancer (fallopian tubes) were used. All the TCGA data were downloaded from the GDSC Broad Institute (http://firebrowse.org). Next, 469 raw MAF files of mutation data were downloaded for ovarian cancer, in which 319 samples have level 3 validated mutations. Samples with level 3 validated mutations were used as the training set to find the miRNAs correlated with genome instability, whereas additional samples with level 2 mutation data were used as an independent validation set. In total, the miRNA microarray data of 570 samples, miRNA-Seq expression of 453 samples, methylation data of 594 samples, clinical information of 591 samples and gene expression of 574 samples were downloaded from the same resource. For the identification of the 17 miRNAs whose expression showed a significant association with the frequency of mutation, the batch-adjusted Agilent 8*15k human miRNA-specific microarray data were used that detects the expression of 723 human miRNAs. Copy number alterations of genes were determined by the GISTIC algorithm and were downloaded together with the results of LOH analysis from the standard analysis of the Broad institute. Germline mutations of BRCA1/2 were obtained from the TCGA publication^32^. Next, 1,078 miRSeq expression profiles and 977 MAF files of TCGA breast cancer were downloaded from the GDSC Broad institute at 07/Jul/2017.

Two datasets, GSE13477 and GSE13525, were downloaded from the GEO database and then were processed using the RMA algorithm in R. After quantile normalization, differential gene expression was analysed by comparing MCF7 cells treated with doxorubicin to control and by comparing 36M2 cells treated with carboplatin to control. Differential expression was determined using the Bioconductor package “limma”, and differentially expressed genes were defined by FDR < 0.05.

To identify the tumours with epigenetically silenced BRCA1/2, K-means consensus clustering combining DNA methylation data and gene expression were used to separate silenced tumours and other tumours. In other words, only tumours with hyper-methylation and downregulated gene expression were considered as epigenetically silenced. The definition of the pathways commonly altered in ovarian cancer and the sensitivity to platinum of each patient was downloaded from the TCGA publication of ovarian cancer^32^.

The following strategy was used to select two groups of patients with the same distribution of score or number of mutations. We first ordered samples by the number of mutations (scores). If the rank of a sample from the high-score (high-mutation) group was followed by the rank of a sample from the low-score (low-mutation) group, the two samples were selected and divided into the high-score (high-mutation) group and low-score (low-mutation) group separately. Samples that did not meet this criterion were discarded. This resulted in two equal groups of patients with different scores (numbers of mutations) but similar mutation numbers (scores), which we further evaluated by the Mann-Whitney U test.

This study was approved by the Ethics Committee of the Harbin Medical University. Informed consent was obtained. All methods were performed in accordance with the relevant guidelines and regulations.

### Statistical analysis

The association between the number of mutations or LOH and BRCA1/2 deficiency was evaluated by the non-parameter Wilcoxon rank-sum test or Mann-Whitney U test. To minimize the retrospective optimization, we deliberately used a simple strategy to develop the signature by assigning a point for a miRNA if a tumour shows a higher or lower than median expression of that miRNA. To be consistent, the miRNAs associated with genome instability were identified by partitioning tumours into two groups according to the median value of miRNA expression, and then evaluated the difference of mutation frequency between the two groups using the Mann-Whitney U test. Survival analyses were conducted by the Kaplan-Meier method using the log-rank test. The univariate and multivariate analyses with Cox regression were used to determine the proportional hazards. All statistical analyses were performed using R software. All the statistical analyses in this study were two-sided, or otherwise specified. Significance was defined when p < 0.05.

### Ethical approval and informed consent

This study was approved by the Ethics Committee of the Harbin Medical University, and informed consent was obtained.

### Data availability

All data generated or analysed during this study are included in this published article (and its Supplementary Information files).

## Electronic supplementary material

Supplementary Information

Supplementary Dataset 2
