# Supplementary material for: The expression of miRNAs is associated with tumour genome instability and predicts the outcome of ovarian cancer patients treated with platinum agents
Source: Sci Rep. 2017 Nov 7;7:14736. doi: 10.1038/s41598-017-12259-w (PMC5677022; doi:10.1038/s41598-017-12259-w)
Supplement: Supplementary file 1 — Supplementary Information [file 41598_2017_12259_MOESM1_ESM.pdf]

The expression of miRNAs is associated with tumour genome instability and predicts the outcome of ovarian cancer patients treated with platinum agents

Tianzhen Wang[1,+], Guangyu Wang[2,+], Xiaoxiao Zhang[3,+], Di Wu[4], Lei Yang[3], Xiaobo Li[1,*], Dapeng Hao[1,*]

1. Department of Pathology, Harbin Medical University, Harbin 150081, China

2. Department of Gastrointestinal Medical Oncology, the Affiliated Tumour Hospital of Harbin Medical University, Harbin 150081, China

3. College of Bioinformatics Science and Technology, Harbin Medical University, Harbin 150081 China
4. Department of Obstetrics and Gynecology, First Affiliated Hospital of Harbin Medical University, Harbin 150001, China.

+: These authors contributed equally to this work.

*: Corresponding:

Xiaobo Li, Email: lixiaobo@ems.hrbmu.edu.cn;

DapengHao, Email: haodapeng@ems.hrbmu.edu.cn.

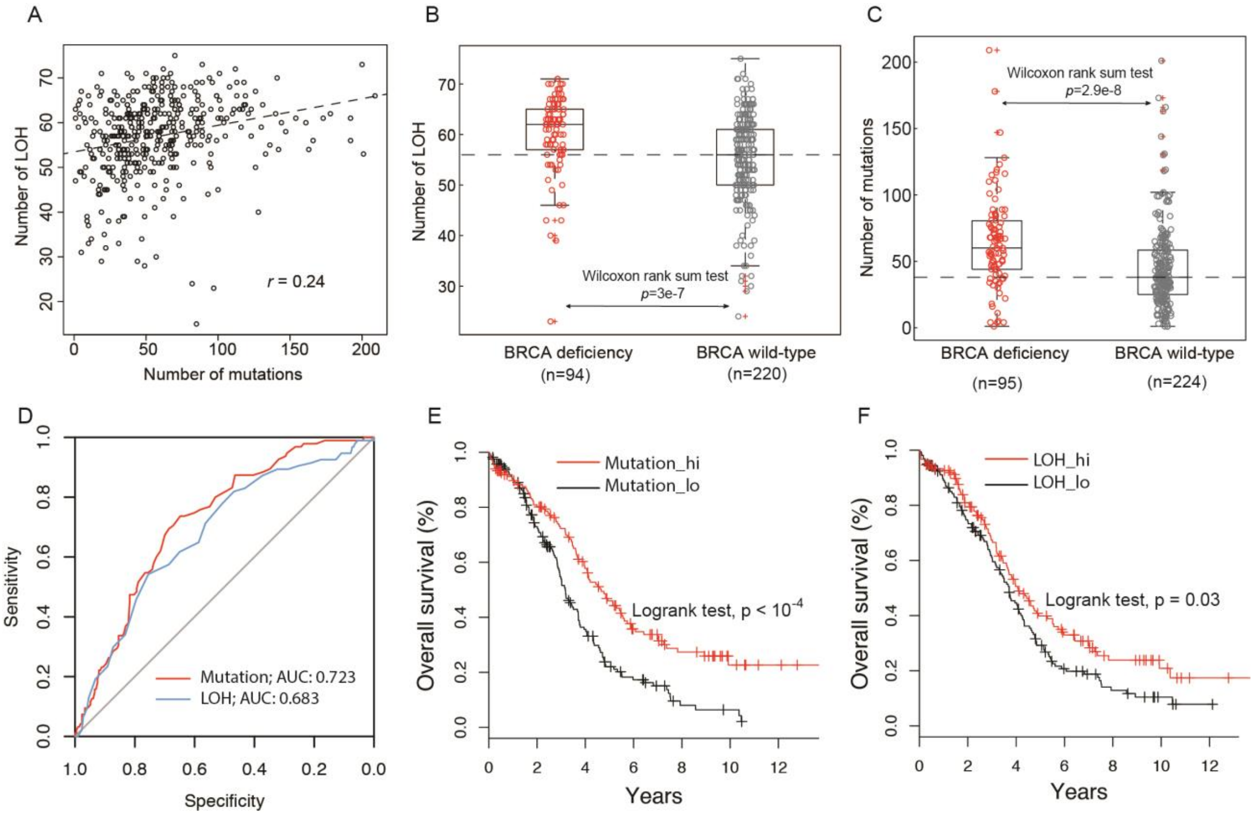

Supplementary Figure 1. Defining the appropriate measures for genome instability in ovarian cancer. (**A**) The number of mutations is correlated with the number of LOH in ovarian cancer (r=0.24). (**B**) The number of LOH is significantly correlated with *BRCA1/2* deficiency (including germline or somatic mutations and epigenetic siliencing events of *BRCA1/2*) in ovarian cancer genome. (**C**) The number of mutations is significantly correlated with *BRCA1/2* deficiency in ovarian cancer genome (p < 0.01). (**D**) ROC curves showing the predictive performance using the frequency of mutation and LOH to identify BRCA deficient tumors. (**E**) Kaplan-Meier overall survival curves showing the association between mutation freuqncy (high vs. low) and clinical outcome of ovarian cancer. (**F**) Kaplan-Meier overall survival curves showing the association between LOH freuqncy (high vs. low) and clinical outcome of ovarian cancer.

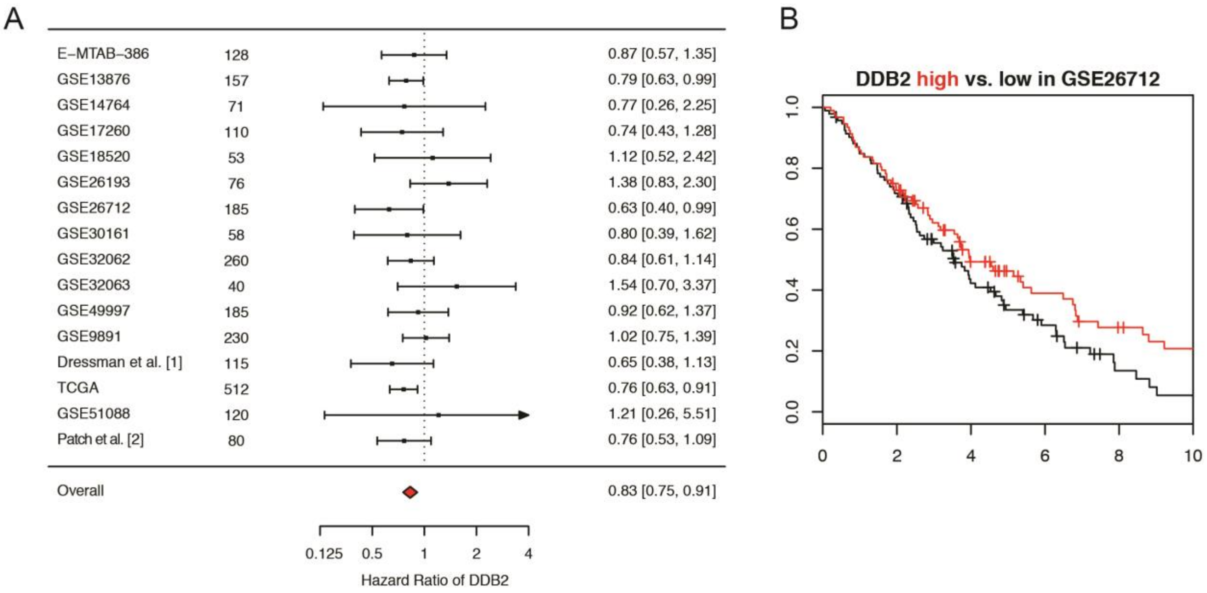

Supplementary Figure 2. The prognostic value of platinum responsive genes. (**A**) An example of the meta-analytic strategy. The prognostic value of gene DDB2 in ovarian cancer is estimated by leveraging 16 transcriptome datasets. Segments show 95% CIs, and the red diamonds show the fixed-effects meta-analysis summaries of HRs over all the datasets.We repeated the same strategy on the 80 platinum responsive genes (Supplementary Table 2). (**B**) Kaplan-Meier overall survival curves showing the association between expression of DDB2 and clinical outcome of ovarian cancer in the dataset GSE26712 (logrank p = 0.07).

References

1. Dressman HK, Berchuck A, Chan G, Zhai J, Bild A, Sayer R, et al. An integrated genomic-based approach to individualized treatment of patients with advanced-stage ovarian cancer. Journal of clinical oncology : official journal of the American Society of Clinical Oncology 2007;25(5):517-25 doi 10.1200/JCO.2006.06.3743.
2. Patch AM, Christie EL, Etemadmoghadam D, Garsed DW, George J, Fereday S, et al. Whole-genome characterization of chemoresistant ovarian cancer. Nature 2015;521(7553):489-94 doi 10.1038/nature14410.

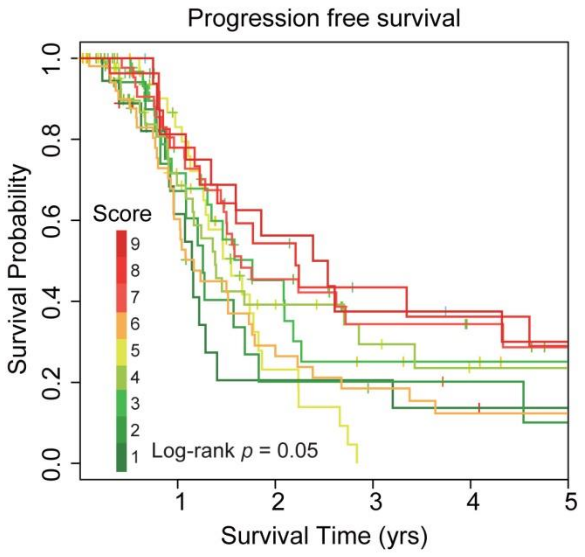

Supplementary Figure 3. Favorable progression free survival is associated with increasing value of score based on the expression of the 10 miRNAs.

Supplementary table 1. Literatures supporing miRNA_targets

microRNA	Target	Pathway	PMID	Reference
miR-100	ATM	ATM	20869334	[1]
miR-101	ATM	ATM	20617180	[2]
miR-18	ATM	ATM	21980462	[3]
miR-421	ATM	ATM	20080624	[4]
miR-146	BRCA1	HR	21472990	[5]
miR-182	BRCA1	HR	21195000	[6]
miR-195	CHEK1	ATM	21778430	[7]
miR-497	CHEK1	ATM	24464213	[8]
miR-182	CHEK2	ATM	21195000	[6]
miR-106	H2AX	ATM	19597473	[9]
miR-138	H2AX	ATM	21693595	[10]
miR-17	H2AX	ATM	19597473	[9]
miR-20	H2AX	ATM	19597473	[9]
miR-215	H2AX	ATM	19597473	[9]
miR-24	H2AX	ATM	19377482	[11]
miR-93	H2AX	ATM	19597473	[9]
miR-193	mcl-1	ATM	23546867	[12]
miR-16	UNG2	BER	23228472	[13]
miR-199	UNG2	BER	23228472	[13]
miR-34	UNG2	BER	23228472	[13]
miR-361	VEGF	BER	24865854	[14]
let-7	E2F2	DDS	20418948	[15]
miR-185	ATR	DDS	23807228	[16]
miR-106	E2F1	DDS	17135268	[17]

miR-17	E2F1	DDS	18836483	[18]
miR-20	E2F1	DDS	18836483	[18]
miR-34	p53	DDS	24884974	[19]
miR-449	E2F1	DDS	19833767	[20]
miR-93	E2F1	DDS	17135268	[17]
miR-106	p21	DDS	20878953	[21]
miR-17	p21	DDS	20190813	[22]
miR-192	p21	DDS	19074875	[23]
miR-215	p21	DDS	20190813	[22]
miR-93	p21	DDS	20190813	[22]
miR-181	p27	DDS	19273599	[24]
miR-221	p27	DDS	17627278	[25]
miR-222	p27	DDS	17627278	[25]
miR-125	p53	DDS	19293287	[26]
miR-25	p53	DDS	20542001	[27]
miR-30	p53	DDS	20542001	[27]
miR-491	p53	DDS	23519249	[28]
miR-504	p53	DDS	20542001	[27]
miR-98	p53	DDS	21880462	[29]
miR-221	p57	DDS	18521080	[30]
miR-222	p57	DDS	23447020	[31]
miR-302	p63	DDS	19342891	[32]
miR-92	p63	DDS	19608627	[33]
miR-29	p85	DDS	19079265	[34]
miR-502	p53	DDS	24374662	[35]
miR-301	p63	DDS	24398967	[36]
miR-373	RAD23B	HR	19141645	[37]

miR-96	RAD51	HR	22761336	[38]
miR-210	RAD52	HR	19141645	[37]
miR-373	RAD52	HR	19141645	[37]
miR-34	SIRT1	HR	18755897	[39]
miR-151	RhoGDIA	HR	20305651	[40]
miR-301	PTEN	HR	21393507	[41]
miR-18	MDM2	MMR	23365201	[42]
miR-192	MDM2	MMR	20951946	[43]
miR-194	MDM2	MMR	20951946	[43]
miR-215	MDM2	MMR	20951946	[43]
miR-25	MDM2	MMR	22431589	[44]
miR-32	MDM2	MMR	22431589	[44]
miR-661	MDM2	MMR	24141721	[45]
miR-155	MLH1	MMR	20351277	[46]
miR-155	MSH2	MMR	20351277	[46]
miR-21	MSH2	MMR	21078976	[47]
miR-21	MSH6	MMR	21078976	[47]
miR-28	Nm23-H1	MMR	22240480	[48]
miR-324	GLI1	MMR	24706306	[49]
miR-124	CDK2	NER	19404929	[50]
miR-302	CDK2	NER	21062975	[51]
miR-372	CDK2	NER	21646351	[52]
miR-885	CDK2	NER	21233845	[53]
miR-124	CDK6	NER	18607543	[54]
miR-29	CDK6	NER	20086245	[55]
miR-449	CDK6	NER	19833767	[20]
miR-521	CSA	NER	18668526	[56]

miR-192	ERCC3	NER	21672525	[57]
miR-192	ERCC4	NER	21672525	[57]
miR-23	FANCG	NER	21750350	[58]
miR-96	REV1	NER	22761336	[38]
miR-211	MMP-9	NER	23183822	[59]
miR-505	AKT3	NHEJ	22051041	[60]
let-7	c-myc	OTHER	19574298	[61]
miR-15	BCL2	OTHER	16166262	[62]
miR-16	BCL2	OTHER	16166262	[62]
miR-205	BCL2	OTHER	23612742	[63]
miR-34	BCL2	OTHER	17656095	[64]
miR-372	CCNA1	OTHER	21646351	[52]
miR-16	Cdc25A	OTHER	19536137	[65]
miR-21	Cdc25A	OTHER	19738433	[66]
miR-322	Cdc25A	OTHER	20462953	[67]
miR-424	Cdc25A	OTHER	20462953	[67]
miR-449	Cdc25A	OTHER	19833767	[20]
miR-503	Cdc25A	OTHER	20462953	[67]
miR-29	CDC42	OTHER	19079265	[34]
miR-145	c-myc	OTHER	19202062	[68]
miR-15	Cyclin D	OTHER	18931683	[69]
miR-16	Cyclin D	OTHER	18931683	[69]
miR-15	Cyclin E	OTHER	19117988	[70]
miR-16	Cyclin E	OTHER	19944013	[71]
miR-29	Cyclin E	OTHER	21551130	[72]
miR-122	Cyclin G1	OTHER	19584283	[73]
miR-101	DNA-PKcs	OTHER	20617180	[2]

miR-128	Wee1	OTHER	20668041	[74]
miR-155	Wee1	OTHER	20668041	[74]
miR-195	Wee1	OTHER	22190509	[75]
miR-516	Wee1	OTHER	20668041	[74]
miR-16	Wip1	OTHER	20668064	[76]
miR-29	Wip1	OTHER	21522133	[77]
miR-320	SMAR1	OTHER	23876508	[78]
miR-28	Cyclin D	OTHER	22240480	[48]
miR-25	REV3L	TLS	22349819	[79]
miR-32	REV3L	TLS	22349819	[79]
miR-26	Cyclin-D	OTHER	19524505	[80]
miR-26	Cyclin-E	OTHER	19524505	[80]
miR-122	Cyclin-G	OTHER	17616664	[81]
miR-122	BCL-w	OTHER	18692484	[82]
miR-195	Cyclin-D	OTHER	19441017	[83]
miR-195	CDK6	NER	19441017	[83]
miR-195	E2F3	DDS	19441017	[83]
miR-221	PTEN	HR	19962668	[84]
miR-222	PTEN	HR	19962668	[84]
miR-29	mcl-1	ATM	17404574	[85]
miR-101	mcl-1	ATM	19155302	[86]
miR-127	BCL6	OTHER	18645025	[87]
miR-28	BRCA1	ATM	18645025	[87]
miR-22	CDK6	NER	21502362	[88]
miR-22	SIRT1	HR	21502362	[88]
miR-133	MMP-9	NER	24714873	[89]
miR-126	VEGF	BER	19223090	[90]

miR-718	VEGF	BER	24815691	[91]
miR-200	VEGF	BER	21357793	[92]
miR-145	AKT3	NHEJ	24781864	[93]
miR-489	AKT3	NHEJ	24686007	[94]
miR-17	AKT1	NHEJ	24658544	[95]
miR-20	AKT1	NHEJ	24658544	[95]
miR-122	AKT3	NHEJ	24244539	[96]
miR-29	AKT3	NHEJ	23764849	[97]
miR-133	GLI1	MMR	24443799	[98]
miR-202	GLI1	MMR	23936094	[99]

Reference

[1]. Ng, W. L.,Yan, D.,Zhang, X., etc. Over-expression of miR-100 is responsible for the low-expression of ATM in the human glioma cell line: M059J[J]. In DNA repair, Nov 10, 2010, 9 (11): 1170-5.

[2]. Yan, D.,Ng, W. L.,Zhang, X., etc. Targeting DNA-PKcs and ATM with miR-101 sensitizes tumors to radiation[J]. In PloS one, 2010, 5 (7): e11397.

[3]. Song, L.,Lin, C.,Wu, Z., etc. miR-18a impairs DNA damage response through downregulation of ataxia telangiectasia mutated (ATM) kinase[J]. In PloS one, 2011, 6 (9): e25454.

[4]. Hu, H.,Du, L.,Nagabayashi, G., etc. ATM is down-regulated by N-Myc-regulated microRNA-421[J]. In Proceedings of the National Academy of Sciences of the United States of America, Jan 26, 2010, 107 (4): 1506-11.

[5]. Garcia, A. I.,Buisson, M.,Bertrand, P., etc. Down-regulation of BRCA1 expression by miR-146a and miR-146b-5p in triple negative sporadic breast cancers[J]. In EMBO molecular medicine, May, 2011, 3 (5): 279-90.

[6]. Moskwa, P.,Buffa, F. M.,Pan, Y., etc. miR-182-mediated downregulation of BRCA1 impacts DNA repair and sensitivity to PARP inhibitors[J]. In Molecular cell, Jan 21, 2011, 41 (2): 210-20.

[7]. Porrello, E. R.,Johnson, B. A.,Aurora, A. B., etc. MiR-15 family regulates postnatal mitotic arrest of cardiomyocytes[J]. In Circulation research, Sep 2, 2011, 109 (6): 670-9.

[8]. Xie, Y.,Wei, R. R.,Huang, G. L., etc. Checkpoint kinase 1 is negatively regulated by miR-497 in hepatocellular carcinoma[J]. In Medical oncology (Northwood, London, England), Mar, 2014, 31 (3): 844.

[9]. Ebi, H.,Sato, T.,Sugito, N., etc. Counterbalance between RB inactivation and miR-17-92 overexpression in reactive oxygen species and DNA damage induction in lung cancers[J]. In Oncogene, Sep 24, 2009, 28 (38): 3371-9.

[10]. Wang, Y.,Huang, J. W.,Li, M., etc. MicroRNA-138 modulates DNA damage response by repressing histone H2AX expression[J]. In Molecular cancer research : MCR, Aug, 2011, 9 (8): 1100-11.

[11]. Lal, A.,Pan, Y.,Navarro, F., etc. miR-24-mediated downregulation of H2AX suppresses DNA repair in terminally differentiated blood cells[J]. In Nature structural & molecular biology, May, 2009, 16 (5): 492-8.

[12]. Kwon, J. E.,Kim, B. Y.,Kwak, S. Y., etc. Ionizing radiation-inducible microRNA miR-193a-3p induces apoptosis by directly targeting Mcl-1[J]. In Apoptosis : an international journal on programmed cell death, Jul, 2013, 18 (7): 896-909.

[13]. Hegre, S. A.,Saetrom, P.,Aas, P. A., etc. Multiple microRNAs may regulate the DNA repair enzyme uracil-DNA glycosylase[J]. In DNA repair, Jan 1, 2013, 12 (1): 80-6.

[14]. Wang, H. W.,Lo, H. H.,Chiu, Y. L., etc. Dysregulated miR-361-5p/VEGF Axis in the Plasma and Endothelial Progenitor Cells of Patients with Coronary Artery Disease[J]. In PloS one, 2014, 9 (5): e98070.

[15]. Dong, Q.,Meng, P.,Wang, T., etc. MicroRNA let-7a inhibits proliferation of human prostate cancer cells in vitro and in vivo by targeting E2F2 and CCND2[J]. In PloS one, 2010, 5 (4): e10147.

[16]. Wang, J.,He, J.,Su, F., etc. Repression of ATR pathway by miR-185 enhances radiation-induced apoptosis and proliferation inhibition[J]. In Cell death & disease, 2013, 4 e699.

[17]. Woods, K.,Thomson, J. M.,Hammond, S. M. Direct regulation of an oncogenic micro-RNA cluster by E2F transcription factors[J]. In The Journal of biological chemistry, Jan 26, 2007, 282 (4): 2130-4.

[18]. Pickering, M. T.,Stadler, B. M.,Kowalik, T. F. miR-17 and miR-20a temper an E2F1-induced G1 checkpoint to regulate cell cycle progression[J]. In Oncogene, Jan 8, 2009, 28 (1): 140-5.

[19]. Zhang, D. G.,Zheng, J. N.,Pei, D. S. P53/microRNA-34-induced metabolic regulation: new opportunities in anticancer therapy[J]. In Molecular cancer, 2014, 13 (1): 115.

[20]. Yang, X.,Feng, M.,Jiang, X., etc. miR-449a and miR-449b are direct transcriptional targets of E2F1 and negatively regulate pRb-E2F1 activity through a feedback loop by targeting CDK6 and CDC25A[J]. In Genes & development, Oct 15, 2009, 23 (20): 2388-93.

[21]. Li, B.,Shi, X. B.,Nori, D., etc. Down-regulation of microRNA 106b is involved in p21-mediated cell cycle arrest in response to radiation in prostate cancer cells[J]. In The Prostate, May, 2011, 71 (6): 567-74.

[22]. Wu, S.,Huang, S.,Ding, J., etc. Multiple microRNAs modulate p21Cip1/Waf1 expression by directly targeting its 3' untranslated region[J]. In Oncogene, Apr 15, 2010, 29 (15): 2302-8.

[23]. Braun, C. J.,Zhang, X.,Savelyeva, I., etc. p53-Responsive micrornas 192 and 215 are capable of inducing cell cycle arrest[J]. In Cancer research, Dec 15, 2008, 68 (24): 10094-104.

[24]. Cuesta, R.,Martinez-Sanchez, A.,Gebauer, F. miR-181a regulates cap-dependent translation of p27(kip1) mRNA in myeloid cells[J]. In Molecular and cellular biology, May, 2009, 29 (10): 2841-51.

[25]. le Sage, C.,Nagel, R.,Egan, D. A., etc. Regulation of the p27(Kip1) tumor suppressor by miR-221 and miR-222 promotes cancer cell proliferation[J]. In The EMBO journal, Aug 8, 2007, 26 (15): 3699-708.

[26]. Le, M. T.,Teh, C.,Shyh-Chang, N., etc. MicroRNA-125b is a novel negative regulator of p53[J]. In Genes & development, Apr 1, 2009, 23 (7): 862-76.

[27]. Hu, W.,Chan, C. S.,Wu, R., etc. Negative regulation of tumor suppressor p53 by microRNA miR-504[J]. In Molecular cell, Jun 11, 2010, 38 (5): 689-99.

[28]. Guo, R.,Wang, Y.,Shi, W. Y., etc. MicroRNA miR-491-5p targeting both TP53 and Bcl-XL induces cell apoptosis in SW1990 pancreatic cancer cells through mitochondria mediated pathway[J]. In Molecules (Basel, Switzerland), 2012, 17 (12): 14733-47.

[29]. Zhang, S.,Zhang, C.,Li, Y., etc. miR-98 regulates cisplatin-induced A549 cell death by inhibiting TP53 pathway[J]. In Biomedicine & pharmacotherapy = Biomedecine & pharmacotherapie, Sep, 2011, 65 (6): 436-42.

[30]. Fornari, F.,Gramantieri, L.,Ferracin, M., etc. MiR-221 controls CDKN1C/p57 and CDKN1B/p27 expression in human hepatocellular carcinoma[J]. In Oncogene, Sep 25, 2008, 27 (43): 5651-61.

[31]. Zhang, J.,Zhou, Y.,Ma, L., etc. The alteration of miR-222 and its target genes in nickel-induced tumor[J]. In Biological trace element research, May, 2013, 152 (2): 267-74.

[32]. Scheel, A. H.,Beyer, U.,Agami, R., etc. Immunofluorescence-based screening identifies germ cell associated microRNA 302 as an antagonist to p63 expression[J]. In Cell cycle (Georgetown, Tex.), May 1, 2009, 8 (9): 1426-32.

[33]. Manni, I.,Artuso, S.,Careccia, S., etc. The microRNA miR-92 increases proliferation of myeloid cells and by targeting p63 modulates the abundance of its isoforms[J]. In FASEB journal : official publication of the Federation of American Societies for Experimental Biology, Nov, 2009, 23 (11): 3957-66.

[34]. Park, S. Y.,Lee, J. H.,Ha, M., etc. miR-29 miRNAs activate p53 by targeting p85 alpha and CDC42[J]. In Nature structural & molecular biology, Jan, 2009, 16 (1): 23-9.

[35]. Yang, S.,Guo, H.,Wei, B., etc. Association of miR-502-binding site single nucleotide polymorphism in the 3'-untranslated region of SET8 and TP53 codon 72 polymorphism with non-small cell lung cancer in Chinese population[J]. In Acta biochimica et biophysica Sinica, Feb, 2014, 46 (2): 149-54.

[36]. Funamizu, N.,Lacy, C. R.,Parpart, S. T., etc. MicroRNA-301b promotes cell invasiveness through targeting TP63 in pancreatic carcinoma cells[J]. In International journal of oncology, Mar, 2014, 44 (3): 725-34.

[37]. Crosby, M. E.,Kulshreshtha, R.,Ivan, M., etc. MicroRNA regulation of DNA repair gene expression in hypoxic stress[J]. In Cancer research, Feb 1, 2009, 69 (3): 1221-9.

[38]. Wang, Y.,Huang, J. W.,Calses, P., etc. MiR-96 downregulates REV1 and RAD51 to promote cellular sensitivity to cisplatin and PARP inhibition[J]. In Cancer research, Aug 15, 2012, 72 (16): 4037-46.

[39]. Yamakuchi, M.,Ferlito, M.,Lowenstein, C. J. miR-34a repression of SIRT1 regulates apoptosis[J]. In Proceedings of the National Academy of Sciences of the United States of America, Sep 9, 2008, 105 (36): 13421-6.

[40]. Ding, J.,Huang, S.,Wu, S., etc. Gain of miR-151 on chromosome 8q24.3 facilitates tumour cell migration and spreading through downregulating RhoGDIA[J]. In Nature cell biology, Apr, 2010, 12 (4): 390-9.

[41]. Shi, W.,Gerster, K.,Alajez, N. M., etc. MicroRNA-301 mediates proliferation and invasion in human breast cancer[J]. In Cancer research, Apr 15, 2011, 71 (8): 2926-37.

[42]. Dar, A. A.,Majid, S.,Rittsteuer, C., etc. The role of miR-18b in MDM2-p53 pathway signaling and melanoma progression[J]. In Journal of the National Cancer Institute, Mar 20, 2013, 105 (6): 433-42.

[43]. Pichiorri, F.,Suh, S. S.,Rocci, A., etc. Downregulation of p53-inducible microRNAs 192, 194, and 215 impairs the p53/MDM2 autoregulatory loop in multiple myeloma development[J]. In Cancer cell, Oct 19, 2010, 18 (4): 367-81.

[44]. Suh, S. S.,Yoo, J. Y.,Nuovo, G. J., etc. MicroRNAs/TP53 feedback circuitry in glioblastoma multiforme[J]. In Proceedings of the National Academy of Sciences of the United States of America, Apr 3, 2012, 109 (14): 5316-21.

[45]. Hoffman, Y.,Bublik, D. R.,Pilpel, Y., etc. miR-661 downregulates both Mdm2 and Mdm4 to activate p53[J]. In Cell death and differentiation, Feb, 2014, 21 (2): 302-9.

[46]. Valeri, N.,Gasparini, P.,Fabbri, M., etc. Modulation of mismatch repair and genomic stability by miR-155[J]. In Proceedings of the National Academy of Sciences of the United States of America, Apr 13, 2010, 107 (15): 6982-7.

[47]. Valeri, N.,Gasparini, P.,Braconi, C., etc. MicroRNA-21 induces resistance to 5-fluorouracil by down-regulating human DNA MutS homolog 2 (hMSH2)[J]. In Proceedings of the National Academy of Sciences of the United States of America, Dec 7, 2010, 107 (49): 21098-103.

[48]. Almeida, M. I.,Nicoloso, M. S.,Zeng, L., etc. Strand-specific miR-28-5p and miR-28-3p have distinct effects in colorectal cancer cells[J]. In Gastroenterology, Apr, 2012, 142 (4): 886-896 e9.

[49]. Xu, H. S.,Zong, H. L.,Shang, M., etc. MiR-324-5p inhibits proliferation of glioma by target regulation of GLI1[J]. In European review for medical and pharmacological sciences, Mar, 2014, 18 (6): 828-32.

[50]. Nakamachi, Y.,Kawano, S.,Takenokuchi, M., etc. MicroRNA-124a is a key regulator of proliferation and monocyte chemoattractant protein 1 secretion in fibroblast-like synoviocytes from patients with rheumatoid arthritis[J]. In Arthritis and rheumatism, May, 2009, 60 (5): 1294-304.

[51]. Lin, S. L.,Chang, D. C.,Ying, S. Y., etc. MicroRNA miR-302 inhibits the tumorigenecity of human pluripotent stem cells by coordinate suppression of the CDK2 and CDK4/6 cell cycle pathways[J]. In Cancer research, Nov 15, 2010, 70 (22): 9473-82.

[52]. Tian, R. Q.,Wang, X. H.,Hou, L. J., etc. MicroRNA-372 is down-regulated and targets cyclin-dependent kinase 2 (CDK2) and cyclin A1 in human cervical cancer, which may contribute to tumorigenesis[J]. In The Journal of biological chemistry, Jul 22, 2011, 286 (29): 25556-63.

[53]. Afanasyeva, E. A.,Mestdagh, P.,Kumps, C., etc. MicroRNA miR-885-5p targets CDK2 and MCM5, activates p53 and inhibits proliferation and survival[J]. In Cell death and differentiation, Jun, 2011, 18 (6): 974-84.

[54]. Pierson, J.,Hostager, B.,Fan, R., etc. Regulation of cyclin dependent kinase 6 by microRNA 124 in medulloblastoma[J]. In Journal of neuro-oncology, Oct, 2008, 90 (1): 1-7.

[55]. Zhao, J. J.,Lin, J.,Lwin, T., etc. microRNA expression profile and identification of miR-29 as a prognostic marker and pathogenetic factor by targeting CDK6 in mantle cell lymphoma[J]. In Blood, Apr 1, 2010, 115 (13): 2630-9.

[56]. Josson, S.,Sung, S. Y.,Lao, K., etc. Radiation modulation of microRNA in prostate cancer cell lines[J]. In The Prostate, Nov 1, 2008, 68 (15): 1599-606.

[57]. Xie, Q. H.,He, X. X.,Chang, Y., etc. MiR-192 inhibits nucleotide excision repair by targeting ERCC3 and ERCC4 in HepG2.2.15 cells[J]. In Biochemical and biophysical research communications, Jul 8, 2011, 410 (3): 440-5.

[58]. Tsai, Y. S.,Lin, C. S.,Chiang, S. L., etc. Areca nut induces miR-23a and inhibits repair of DNA double-strand breaks by targeting FANCG[J]. In Toxicological sciences : an official journal of the Society of Toxicology, Oct, 2011, 123 (2): 480-90.

[59]. Asuthkar, S.,Velpula, K. K.,Chetty, C., etc. Epigenetic regulation of miRNA-211 by MMP-9 governs glioma cell apoptosis, chemosensitivity and radiosensitivity[J]. In Oncotarget, Nov, 2012, 3 (11): 1439-54.

[60]. Yamamoto, Y.,Yoshioka, Y.,Minoura, K., etc. An integrative genomic analysis revealed the relevance of microRNA and gene expression for drug-resistance in human breast cancer cells[J]. In Molecular cancer, 2011, 10 135.

[61]. Kim, H. H.,Kuwano, Y.,Srikantan, S., etc. HuR recruits let-7/RISC to repress c-Myc expression[J]. In Genes & development, Aug 1, 2009, 23 (15): 1743-8.

[62]. Cimmino, A.,Calin, G. A.,Fabbri, M., etc. miR-15 and miR-16 induce apoptosis by targeting BCL2[J]. In Proceedings of the National Academy of Sciences of the United States of America, Sep 27, 2005, 102 (39): 13944-9.

[63]. Verdoodt, B.,Neid, M.,Vogt, M., etc. MicroRNA-205, a novel regulator of the anti-apoptotic protein Bcl2, is downregulated in prostate cancer[J]. In International journal of oncology, Jul, 2013, 43 (1): 307-14.

[64]. Bommer, G. T.,Gerin, I.,Feng, Y., etc. p53-mediated activation of miRNA34 candidate tumor-suppressor genes[J]. In Current biology : CB, Aug 7, 2007, 17 (15): 1298-307.

[65]. Pothof, J.,Verkaik, N. S.,van, I. W., etc. MicroRNA-mediated gene silencing modulates the UV-induced DNA-damage response[J]. In The EMBO journal, Jul 22, 2009, 28 (14): 2090-9.

[66]. de Oliveira, P. E.,Zhang, L.,Wang, Z., etc. Hypoxia-mediated regulation of Cdc25A phosphatase by p21 and miR-21[J]. In Cell cycle (Georgetown, Tex.), Oct 1, 2009, 8 (19): 3157-64.

[67]. Sarkar, S.,Dey, B. K.,Dutta, A. MiR-322/424 and -503 are induced during muscle differentiation and promote cell cycle quiescence and differentiation by down-regulation of Cdc25A[J]. In Molecular biology of the cell, Jul 1, 2010, 21 (13): 2138-49.

[68]. Sachdeva, M.,Zhu, S.,Wu, F., etc. p53 represses c-Myc through induction of the tumor suppressor miR-145[J]. In Proceedings of the National Academy of Sciences of the United States of America, Mar 3, 2009, 106 (9): 3207-12.

[69]. Bonci, D.,Coppola, V.,Musumeci, M., etc. The miR-15a-miR-16-1 cluster controls prostate cancer by targeting multiple oncogenic activities[J]. In Nature medicine, Nov, 2008, 14 (11): 1271-7.

[70]. Cohen, E. E.,Zhu, H.,Lingen, M. W., etc. A feed-forward loop involving protein kinase Calpha and microRNAs regulates tumor cell cycle[J]. In Cancer research, Jan 1, 2009, 69 (1): 65-74.

[71]. Wang, F.,Fu, X. D.,Zhou, Y., etc. Down-regulation of the cyclin E1 oncogene expression by microRNA-16-1 induces cell cycle arrest in human cancer cells[J]. In BMB reports, Nov 30, 2009, 42 (11): 725-30.

[72]. Ding, D. P.,Chen, Z. L.,Zhao, X. H., etc. miR-29c induces cell cycle arrest in esophageal squamous cell carcinoma by modulating cyclin E expression[J]. In Carcinogenesis, Jul, 2011, 32 (7): 1025-32.

[73]. Fornari, F.,Gramantieri, L.,Giovannini, C., etc. MiR-122/cyclin G1 interaction modulates p53 activity and affects doxorubicin sensitivity of human hepatocarcinoma cells[J]. In Cancer research, Jul 15, 2009, 69 (14): 5761-7.

[74]. Butz, H.,Liko, I.,Czirjak, S., etc. Down-regulation of Wee1 kinase by a specific subset of microRNA in human sporadic pituitary adenomas[J]. In The Journal of clinical endocrinology and metabolism, Oct, 2010, 95 (10): E181-91.

[75]. He, J. F.,Luo, Y. M.,Wan, X. H., etc. Biogenesis of MiRNA-195 and its role in biogenesis, the cell cycle, and apoptosis[J]. In Journal of biochemical and molecular toxicology, Nov-Dec, 2011, 25 (6): 404-8.

[76]. Zhang, X.,Wan, G.,Mlotshwa, S., etc. Oncogenic Wip1 phosphatase is inhibited by miR-16 in the DNA damage signaling pathway[J]. In Cancer research, Sep 15, 2010, 70 (18): 7176-86.

[77]. Ugalde, A. P.,Ramsay, A. J.,de la Rosa, J., etc. Aging and chronic DNA damage response activate a regulatory pathway involving miR-29 and p53[J]. In The EMBO journal, Jun 1, 2011, 30 (11): 2219-32.

[78]. Mittal, S. P.,Mathai, J.,Kulkarni, A. P., etc. miR-320a regulates erythroid differentiation through MAR binding protein SMAR1[J]. In The international journal of biochemistry & cell biology, Nov, 2013, 45 (11): 2519-29.

[79]. Zhang, S.,Chen, H.,Zhao, X., etc. REV3L 3'UTR 460 T>C polymorphism in microRNA target sites contributes to lung cancer susceptibility[J]. In Oncogene, Jan 10, 2013, 32 (2): 242-50.

[80]. Kota, J.,Chivukula, R. R.,O'Donnell, K. A., etc. Therapeutic microRNA delivery suppresses tumorigenesis in a murine liver cancer model[J]. In Cell, Jun 12, 2009, 137 (6): 1005-17.

[81]. Gramantieri, L.,Ferracin, M.,Fornari, F., etc. Cyclin G1 is a target of miR-122a, a microRNA frequently down-regulated in human hepatocellular carcinoma[J]. In Cancer research, Jul 1, 2007, 67 (13): 6092-9.

[82]. Lin, C. J.,Gong, H. Y.,Tseng, H. C., etc. miR-122 targets an anti-apoptotic gene, Bcl-w, in human hepatocellular carcinoma cell lines[J]. In Biochemical and biophysical research communications, Oct 24, 2008, 375 (3): 315-20.

[83]. Xu, T.,Zhu, Y.,Xiong, Y., etc. MicroRNA-195 suppresses tumorigenicity and regulates G1/S transition of human hepatocellular carcinoma cells[J]. In Hepatology (Baltimore, Md.), Jul, 2009, 50 (1): 113-21.

[84]. Garofalo, M.,Di Leva, G.,Romano, G., etc. miR-221&222 regulate TRAIL resistance and enhance tumorigenicity through PTEN and TIMP3 downregulation[J]. In Cancer cell, Dec 8, 2009, 16 (6): 498-509.

[85]. Mott, J. L.,Kobayashi, S.,Bronk, S. F., etc. mir-29 regulates Mcl-1 protein expression and apoptosis[J]. In Oncogene, Sep 13, 2007, 26 (42): 6133-40.

[86]. Su, H.,Yang, J. R.,Xu, T., etc. MicroRNA-101, down-regulated in hepatocellular carcinoma, promotes apoptosis and suppresses tumorigenicity[J]. In Cancer research, Feb 1, 2009, 69 (3): 1135-42.

[87]. Kovalchuk, O.,Filkowski, J.,Meservy, J., etc. Involvement of microRNA-451 in resistance of the MCF-7 breast cancer cells to chemotherapeutic drug doxorubicin[J]. In Molecular cancer therapeutics, Jul, 2008, 7 (7): 2152-9.

[88]. Xu, D.,Takeshita, F.,Hino, Y., etc. miR-22 represses cancer progression by inducing cellular senescence[J]. In The Journal of cell biology, Apr 18, 2011, 193 (2): 409-24.

[89]. Wu, D.,Pan, H.,Zhou, Y., etc. microRNA-133b downregulation and inhibition of cell proliferation, migration and invasion by targeting matrix metallopeptidase-9 in renal cell carcinoma[J]. In Molecular medicine reports, Jun, 2014, 9 (6): 2491-8.

[90]. Liu, B.,Peng, X. C.,Zheng, X. L., etc. MiR-126 restoration down-regulate VEGF and inhibit the growth of lung cancer cell lines in vitro and in vivo[J]. In Lung cancer (Amsterdam, Netherlands), Nov, 2009, 66 (2): 169-75.

[91]. Leng, R.,Zha, L.,Tang, L. MiR-718 represses VEGF and inhibits ovarian cancer cell progression[J]. In FEBS letters, Jun 5, 2014, 588 (12): 2078-86.

[92]. McArthur, K.,Feng, B.,Wu, Y., etc. MicroRNA-200b regulates vascular endothelial growth factor-mediated alterations in diabetic retinopathy[J]. In Diabetes, Apr, 2011, 60 (4): 1314-23.

[93]. Boufraqech, M.,Zhang, L.,Jain, M., etc. miR-145 suppresses thyroid cancer growth and metastasis and targets AKT3[J]. In Endocrine-related cancer, Apr 29, 2014.

[94]. Wu, H.,Xiao, Z.,Zhang, H., etc. MiR-489 modulates cisplatin resistance in human ovarian cancer cells by targeting Akt3[J]. In Anti-cancer drugs, Mar 29, 2014.

[95]. Yu, Z.,Xu, Z.,Disante, G., etc. miR-17/20 sensitization of breast cancer cells to chemotherapy-induced apoptosis requires Akt1[J]. In Oncotarget, Feb 28, 2014, 5 (4): 1083-90.

[96]. Nassirpour, R.,Mehta, P. P.,Yin, M. J. miR-122 regulates tumorigenesis in hepatocellular carcinoma by targeting AKT3[J]. In PloS one, 2013, 8 (11): e79655.

[97]. Wei, W.,He, H. B.,Zhang, W. Y., etc. miR-29 targets Akt3 to reduce proliferation and facilitate differentiation of myoblasts in skeletal muscle development[J]. In Cell death & disease, 2013, 4 e668.

[98]. Zhao, Y.,Huang, J.,Zhang, L., etc. MiR-133b is frequently decreased in gastric cancer and its overexpression reduces the metastatic potential of gastric cancer cells[J]. In BMC cancer, 2014, 14 34.

[99]. Zhao, Y.,Li, C.,Wang, M., etc. Decrease of miR-202-3p expression, a novel tumor suppressor, in gastric cancer[J]. In PloS one, 2013, 8 (7): e69756.

Supplementary table 3. Platinum response genes.

genes	Freq	pvalue	HR	95CI_L	95CI_U	FDR
**CETN2**	16	6.55E-05	0.85610681	0.79323251	0.92396474	0.00383719
**DDB2**	16	9.84E-05	0.82811098	0.75312267	0.91056586	0.00383719
**ERCC8**	16	0.00021334	1.20665604	1.0924418	1.33281131	0.00554678
**RAD17**	14	0.0013993	1.22008984	1.07992007	1.37845314	0.02728626
**H2AFX**	15	0.00285759	0.89207466	0.82757779	0.96159806	0.03828652
**HLTF**	15	0.00294512	1.1195468	1.03923884	1.20606062	0.03828652
**ERCC1**	15	0.00516841	1.15294936	1.04348766	1.27389357	0.05759091
**UBE2B**	15	0.00595789	1.15503288	1.04227886	1.27998466	0.05808941
**RAD9A**	15	0.01262431	0.87588808	0.78926737	0.97201525	0.1094107
**C17orf70**	13	0.01781211	0.84748503	0.73906987	0.97180375	0.13893444
**BRCA2**	16	0.02126229	1.11547506	1.01642279	1.22418015	0.15076893
**FANCI**	15	0.03140362	0.92855928	0.86794205	0.99341003	0.17859631
**RBBP8**	14	0.03182755	0.925793	0.86285694	0.99331955	0.17859631
**UBE2V2**	14	0.03205575	1.11473167	1.00935341	1.23111159	0.17859631
**FANCA**	15	0.04528682	0.90560708	0.82183257	0.99792126	0.23549149
**PALB2**	16	0.06057379	0.89964902	0.80557477	1.00470917	0.27792681
**TP53BP1**	16	0.05803354	1.12124685	0.99611208	1.26210145	0.27792681
**RAD54L**	16	0.0700907	0.92771627	0.85537468	1.00617601	0.27839393
**BLM**	16	0.06488312	0.92644385	0.85426175	1.0047251	0.27839393
**C19orf40**	13	0.07138306	1.10179646	0.99159792	1.22424161	0.27839393
**POLK**	12	0.08387875	1.0966335	0.98773005	1.21754425	0.31154963
**REV3L**	16	0.09696642	1.07634238	0.9867807	1.17403282	0.34379004
**RAD50**	16	0.12519074	1.07608678	0.97980402	1.18183099	0.42145029
**CHEK2**	11	0.12967701	0.92119596	0.82841034	1.02437399	0.42145029
**RAD54B**	14	0.15263609	0.9490986	0.88352794	1.01953557	0.42520055
**FBXO18**	12	0.14162552	1.09508784	0.97015894	1.23610402	0.42520055
**FANCB**	11	0.14905536	1.09843978	0.96691187	1.24785927	0.42520055
**NBN**	15	0.14906728	1.06075768	0.97908147	1.14924743	0.42520055
**FANCG**	3	0.18728913	0.90284053	0.77559226	1.05096591	0.50374318
**CLSPN**	10	0.20551931	0.93172347	0.835099	1.03952781	0.51784498
**FANCL**	16	0.2257273	0.94798143	0.86949024	1.03355823	0.51784498
**ATRIP**	5	0.22087084	0.86852576	0.69306518	1.08840699	0.51784498
**HUS1**	14	0.21054872	1.06654702	0.96422919	1.17972217	0.51784498
**RAD18**	11	0.21294758	1.08084874	0.95638172	1.22151434	0.51784498
**POLH**	14	0.26355831	0.93945272	0.8420209	1.04815856	0.54409944
**CHEK1**	15	0.26374471	0.95704159	0.88611115	1.03364979	0.54409944
**WDR48**	15	0.26507409	1.06085251	0.95617444	1.1769903	0.54409944
**TOP2A**	16	0.26285499	1.03038382	0.97778735	1.08580952	0.54409944
**BRCA1**	16	0.28178661	1.04349836	0.96564687	1.12762631	0.56357321
**FANCF**	16	0.29669238	0.95103062	0.86544641	1.04507828	0.57855014
**ERCC2**	15	0.31559874	1.06563222	0.94121534	1.20649545	0.60040737
**RAD52**	16	0.34828958	1.04301858	0.95513989	1.13898264	0.60370194
**XPA**	16	0.33620824	1.05769587	0.94343541	1.18579454	0.60370194
**POLI**	16	0.34156359	0.95951198	0.88117119	1.04481768	0.60370194
**TOPBP1**	16	0.32960081	1.04418641	0.95725335	1.13901431	0.60370194

**ATR**	15	0.36584832	0.95912587	0.87617756	1.04992695	0.60715253
**UBE2N**	14	0.35826359	1.04806228	0.94818277	1.15846287	0.60715253
**ERCC5**	16	0.39232984	1.04576436	0.94385525	1.15867671	0.63753598
**ATM**	16	0.42572344	0.96667542	0.88932796	1.05075002	0.66597227
**RNF8**	15	0.44668897	0.96427751	0.87803403	1.05899211	0.66597227
**ERCC3**	16	0.46105772	0.96104964	0.86470305	1.06813131	0.66597227
**DDB1**	16	0.44691025	0.9546292	0.84697358	1.07596853	0.66597227
**FANCC**	16	0.4609922	0.9593408	0.85910441	1.07127233	0.66597227
**XRCC3**	15	0.43155054	0.95368604	0.84739572	1.07330853	0.66597227
**RAD23B**	15	0.48026358	0.97438689	0.90665651	1.04717696	0.68110108
**RAD23A**	15	0.51700041	1.03028754	0.94137447	1.12759847	0.72010772
**FANCM**	9	0.53302936	0.95316624	0.81973571	1.1083156	0.72940859
**XAB2**	16	0.54482562	1.03079423	0.93440982	1.13712068	0.73269652
**TP53**	15	0.57328071	0.98665733	0.94159878	1.03387207	0.75789653
**XPC**	15	0.59870014	0.97098171	0.87012529	1.08352842	0.77831018
**MDC1**	16	0.65707162	0.9795337	0.89407174	1.07316476	0.84018994
**RAD1**	16	0.67176284	0.98096259	0.89751309	1.0721711	0.845121
**ERCC6**	16	0.68378831	1.02461462	0.91146948	1.15180502	0.84659505
**BRIP1**	15	0.74140492	0.9866812	0.91115202	1.06847131	0.85043506
**USP1**	16	0.74089942	0.98706151	0.91373493	1.06627251	0.85043506
**FANCE**	16	0.73722943	0.98308433	0.88983573	1.08610474	0.85043506
**RAD51C**	16	0.73385857	1.0129429	0.94056676	1.09088834	0.85043506
**SHFM1**	12	0.72853796	1.01954817	0.91396145	1.13733296	0.85043506
**FANCD2**	12	0.75758903	1.01640425	0.91662838	1.12704081	0.85640499
**MAD2L2**	12	0.77412735	1.0146967	0.91846403	1.12101221	0.86259905
**REV1**	15	0.78877847	1.01426551	0.9144195	1.12501377	0.86654536
**SHPRH**	12	0.81041584	1.01336934	0.90916679	1.12951489	0.87795049
**MUS81**	16	0.83598101	1.01333702	0.89388811	1.14874771	0.89323999
**MRE11A**	16	0.85117687	1.00826913	0.9251549	1.09885019	0.89718643
**RAD51**	16	0.89212949	0.99447952	0.9180128	1.0773156	0.92781467
**XRCC2**	15	0.91114667	0.99499328	0.91103284	1.08669147	0.93512421
**UBE2I**	16	0.9740197	1.00156294	0.91171708	1.10026272	0.98666931
**ERCC4**	15	0.99519205	0.99967076	0.89813468	1.11268571	0.99519205
**RAD51L1**	0	NA	NA	NA	NA	NA
**RAD51L3**	0	NA	NA	NA	NA	NA
